# Supplementary material for: Profiling system-wide variations and similarities between Rheumatic Heart Disease and Acute Rheumatic Fever–A pilot analysis
Source: PLoS Negl Trop Dis. 2023 Apr 5;17(4):e0011263. doi: 10.1371/journal.pntd.0011263 (PMC10109489; doi:10.1371/journal.pntd.0011263)
Supplement: Attachment — Submitted filename: Reply_to_reviewers_comments.pdf [file pntd.0011263.s004.pdf]

Response to reviewers' comments

We thank the reviewers for their valuable suggestions. We have incorporated all the suggestions put forth and have expanded on the changes incorporated in detail under each comment along with the manuscript page numbers where the respective changes have been added. All responses have been highlighted in yellow.

Reviewer's Responses to Questions
Key Review Criteria Required for Acceptance?
As you describe the new analyses required for acceptance, please consider the following:

Methods
-Are the objectives of the study clearly articulated with a clear testable hypothesis stated?
-Is the study design appropriate to address the stated objectives?
-Is the population clearly described and appropriate for the hypothesis being tested?
-Is the sample size sufficient to ensure adequate power to address the hypothesis being tested?
-Were correct statistical analysis used to support conclusions?
-Are there concerns about ethical or regulatory requirements being met?

Reviewer #1: Manuscript PNTD-22-01009
Profiling system-wide variations and similarities between Rheumatic Heart Disease and Acute Rheumatic Fever
The present article is well written and interesting. The authors describe the identification of a sub network comprising the most significantly and differentially expressed genes and most perturbed pathways in RHD compared to ARF.
They evaluated the transcriptoma of 5 patients presenting ARF and 5 presenting RHD and identified a subnetwork comprising 53 significantly differentially expressed genes and most perturbed pathways in RHD compared 54 to ARF.
Figure 1, describe the overview of the article and is well presented.

Response: We thank the reviewers for reading our manuscript critically and for the encouraging comments.

Reviewer #2: The objectives are stated although only in non-specific terms. The sample size is very small rendering this a pilot analysis which needs to be clearly stated. While that in itself is not problematic there is very limited description of the clinical state of the patients and controls which will markedly influence the results (particularly given the small sample size). There is insufficient description of approaches to deal with multiple testing. Ethical review is described.

Response: We have now described the objectives in more detail and in specific terms (pg 5-6). We have also now clearly stated that it is a pilot study (pg 1, 3, 4, 6, 23). All changes in the manuscript are highlighted in yellow.
More information has now been added about the clinical state of the patients' samples and healthy controls on pg 6-7. Table 1 and Table 2 describe their clinical parameters such as age, gender, height, weight, BMI; and Neutrophils (%), Lymphocytes (%),

Neutrophil-to-Lymphocyte ratio (NLR), ESR (mm/hr), CRP (mg/dl) and ASO (IU/ml) (pg 7, 12).
We have also elaborated on the approach for multiple testing (pg 10 and as described in detail to a comment below)

Results

-Does the analysis presented match the analysis plan?
-Are the results clearly and completely presented?
-Are the figures (Tables, Images) of sufficient quality for clarity?

Reviewer #1: Analysis, are correct, results are clearly and Figure is well presented

Reviewer #2: The results are moderately well presented but there is room for substantial improvement. The figures need to be provided in high resolution format as they are not currently readable. There needs to be much greater clarity of the relationships between the various tests performed i.e. RHD vs ARF, RHD vs HC etc. The Venn diagram provides some help with this but it would be useful to have a flow diagram with references back to this. It should be made much clearer which of the analyses was primary and which were secondary ideally with more stringent thresholds for secondary analyses. The content on the neutrophil-to-lymphocyte ratio seems out of keeping with the wider transcriptomics and further effort should be made to link these together to improve the flow of the manuscript as well as explain whether this work was part of the initial analysis plan.

Response: We thank the reviewer for the suggestion. In the revised manuscript, we have edited Figure 1 to clearly highlight the relationship between the groups and the primary and secondary analyses. The information on the neutrophil-to-lymphocyte ratio is a part of the basic laboratory data that is gathered as a routine workup and has been correlated with the transcriptomic changes. The discussion is now restructured to indicate this clearly (pg 19-20).

Conclusions

-Are the conclusions supported by the data presented?
-Are the limitations of analysis clearly described?
-Do the authors discuss how these data can be helpful to advance our understanding of the topic under study?
-Is public health relevance addressed?

Reviewer #1: Yes for all questions

Reviewer #2: The manuscript would benefit from a clear summary that outlines the key findings in relation to the researchers' hypothesis. A substantially greater proportion of the discussion should be devoted to limitations, particularly in relationship to sample size and the

clinical characteristics of the cohort and how this is might influence the results. At the current sample size the results are extremely preliminary and so the conclusions should be substantially more cautious and so relating the results to pathogenesis is less informative at this stage. A more useful message would be how given these primarily findings 'omics approaches might be useful to the field more broadly and what sample sizes would be necessary for definitive studies.

Response: We agree. We have revised the paragraph at the beginning of the Results section (pg 10-11) to indicate clearly the goals of the primary analysis and the secondary analysis. We have also clearly indicated that this is a pilot study and have added a note on the limitations of the study - primarily due to the small sample size. As suggested, we have added a note on how this can lead to the design of an expanded clinical study to get statistically significant molecular profiles that differ in the two conditions (pg 23).

Editorial and Data Presentation Modifications?

Use this section for editorial suggestions as well as relatively minor modifications of existing data that would enhance clarity. If the only modifications needed are minor and/or editorial, you may wish to recommend “Minor Revision” or “Accept”.

Reviewer #1: None

Reviewer #2: In addition please address the following issues:

Introduction:
Q - Line 110 - correct prevalence in the Noubiap et al paper is 26.1 per 1000

Response: Correction incorporated

Methods:
Q - Line 145 - how were healthy controls selected and what was the justification for using these specific groups?

Response: Although ideally, healthy children would have been the best control group, considering that it was ethically and morally inappropriate to sample healthy children, blood from age-matched children with congenital heart disease (CHD) formed the clinical control group after confirming that they had no clinical, laboratory and echocardiographic evidence of coexisting ARF or RHD.
Additionally, blood draws from healthy individuals over 18 years – (apparently in good health on clinical and laboratory evaluation) served as ‘Healthy Controls’. This is indicated clearly in the Methods section (pg 6-7).

Q - were either the ARF or RHD patients acutely unwell at the time recruitment? if so describe including timing from onset, if not describe time since last episode/recurrence.

Response: The patients with ARF in this study were all hospitalized and as mentioned in the manuscript, the clinical diagnosis was based on modified Jones criteria. The patients with RHD were visiting the outpatient department for follow-up at the time of recruitment and they were all on ARF prophylaxis. None of the patients recruited for

the study had any recurrence in the past six months. We took particular care to exclude RHD cases with acute episodes so as to avoid sampling patients with acute over chronic RHD. This was done with the sole purpose of generating clean data without any ambiguity (pg 7).

- Line 162 - define RIN abbreviations, if RNA integrity number justify why this threshold and not a higher value was used.

Response: RIN does stand for RNA integrity number. RIN values > 7 are generally considered acceptable (Sheng Q et al. 2017). Shah et al. 2019, showed that RIN value >= 6.9 also correlates with a fair A260:280 ratio. Further, >80% mapping to the well-established human reference genome was obtained for RNA sequences with RIN >7. Based on numerous studies that have used this threshold, we, too, use this as a default threshold for proceeding to RNA sequencing. This information has been added to pg 8 of the manuscript.

Sheng Q, Vickers K, Zhao S, Wang J, Samuels DC, Koues O, Shyr Y, Guo Y. Multi-perspective quality control of Illumina RNA sequencing data analysis. Brief Funct Genomics. 2017 Jul 1;16(4):194-204. doi: 10.1093/bfgp/elw035. PMID: 27687708; PMCID: PMC5860075.
Shah SG, Rashid M, Verma T, Ludbe M, Khade B, Gera PB, Gupta S. Establishing a correlation between RIN and A260/280 along with the multivariate evaluation of factors affecting the quality of RNA in cryopreserved cancer bio-specimen. Cell Tissue Bank. 2019 Dec;20(4):489-499. doi: 10.1007/s10561-019-09782-7. Epub 2019 Aug 23. PMID: 31444592.

- Line 177 - given the multi-way comparisons please explain what steps were taken to account for multiple testing

Response: The DESeq2 package, a widely used and accepted R package for differential expression analysis was used in this study. The model fit generated by DESeq estimates a single dispersion parameter for each gene that represents how far the observed count for a sample will be from the mean value of the model, given the size factor and condition groups. The developers of the software mention that "Having a single dispersion parameter for each gene is usually sufficient for analyzing multi-group data, as the final dispersion value will incorporate the within-group variability across all groups." They recommend "users to run samples from all groups together, and then use the contrast argument of the results function to extract comparisons of interest after fitting the model using DESeq". This approach was followed in the study as well where samples from all the groups were run together and the contrast() function was utilized to identify DEGs from each pairwise group comparison. P-value < 0.05 was used as a threshold for all analyses.
Further computation of DEGs with Bonferroni's correction with a threshold of adjusted p-value < 0.05 have also been mentioned.
Reference:
FAQ1-
https://bioconductor.riken.jp/packages/3.6/bioc/vignettes/DESeq2/inst/doc/DESeq2.html

This information has been added to the methods section of the manuscript on pg10.

- Line 183 - for the in-house mining approach please justify use of this approach (as opposed to others) and describe availability of code (so that the work can be reproduced)

Response: The general transcriptomics framework of identifying perturbations in a condition being studied via DEGs alone is highly dependent on sample sizes that skew the statistical significance of the findings. Further, genes do not act alone and are highly regulated by other genes. Thus, a network analysis approach that can identify relevant perturbations at the level of gene interactions is more beneficial. An in-house network mining approach, that provides a global systems perspective of alteration in a condition was hence used to identify the most perturbed gene interactions and pathways (A general version of the code is available in Samaturu et al., 2021, GitHub deposition - https://github.com/NarmadaSambaturu/PathExt, https://github.com/NarmadaSambaturu/EpiTracer). This information has been added to the revised manuscript on pg 9-10. We have found the approach to be very useful in a number of studies (Sambarey et al., 2017, Metri et al., 2017, Thakur et al., 2022, Ravichandran et al., 2021, Banerjee et al., 2022). It has also been cited by other groups, including Hiort et al. 2022 and Agrawal et al., 2022.

This approach has also been bench-marked against other existing network analysis approaches such as WGCNA, ARACNE, and jActiveModules and was found to perform better in (1) enrichment of the gold standard gene sets, (2) comparison to gold standard pathways and (3) recovery of hub genes from the context-specific perturbed network, known to be related to the given condition.WGCNA and ARACNE belong to the broad class of data-driven approaches which do not rely on prior network information. ResponseNet and jActiveModules utilize knowledge-based protein–protein interaction networks and integrate condition-specific transcriptome or proteome data. (Ravichandran et al, 2019).

Ravichandran, S., Chandra, N. Interrogation of genome-wide networks in biology: comparison of knowledge-based and statistical methods. Int J Adv Eng Sci Appl Math 11, 119–137 (2019). https://doi.org/10.1007/s12572-018-0242-9

Sambaturu N, Pusadkar V, Hannenhalli S, Chandra N. PathExt: a general framework for path-based mining of omics-integrated biological networks. Bioinformatics. 2021;37: 1254–1262. doi:10.1093/BIOINFORMATICS/BTAA941

Sambarey A, Devaprasad A, Baloni P, Mishra M, Mohan A, Tyagi P, Singh A, Akshata JS, Sultana R, Buggi S and Chandra N Meta-analysis of host response networks identifies a common core in tuberculosis npj Systems Biology and Applications, 2017, Feb 10;3:4.

Sambarey A, Devaprasad A, Mohan A, Ahmed A, Nayak S, Swaminathan S, D'Souza G, Jesuraj A, Dhar C, Babu S, Vykarnam A, Chandra N. Unbiased Identification of Blood-based Biomarkers for Pulmonary Tuberculosis by Modeling and Mining Molecular Interaction Networks EBioMedicine, 2017, Vol. 15, page 112-126

Metri, R., Mohan, A., Nsengimana, J. et al. Identification of a gene signature for discriminating metastatic from primary melanoma using a molecular interaction network approach. Sci Rep 7, 17314, 2017. https://doi.org/10.1038/s41598-017-17330-0

Thakur C., Tripathi A., Ravichandran S., Shivananjaiah A., Chakraborthy A., Varadappa S., Chikkavenkatappa N., Nagarajan D., Lakshminarasimhaiah S., Singh A. and Chandra N. A new blood-based RNA signature (R9), for monitoring effectiveness of tuberculosis treatment in a South Indian longitudinal cohort iScience, 2022, 25, 103745.

Ravichandran S., Banerjee U., DR Devi G., Kandukuru R., Thakur C., Chakravorthy D., Kithiganahalli B., Singh A. and Chandra N. VB10, a new blood biomarker for differential diagnosis and recovery monitoring of acute viral and bacterial infections EBioMedicine, 2021, 67, 103352.

Banerjee U., Rao P., Reddy M., Hussain M., Chunchanur S., Ambica R., Singh A. and Chandra N. A 9-gene biomarker panel identifies bacterial coinfections in culture-negative COVID-19 cases. Molecular Omics, 2022.

Pauline Hiort, Julian Hugo, Justus Zeinert, Nataniel Müller, Spoorthi Kashyap, Jagath C Rajapakse, Francisco Azuaje, Bernhard Y Renard, Katharina Baum, DrDimont: explainable drug response prediction from differential analysis of multi-omics networks, Bioinformatics, Volume 38, Issue Supplement_2, September 2022, Pages ii113–ii119, https://doi.org/10.1093/bioinformatics/btac477

Agrawal, P., Sambaturu, N., Olgun, G. and Hannenhalli, S., 2022. A path-based analysis of infected cell line and COVID-19 patient transcriptome reveals novel potential targets and drugs against SARS-CoV-2. Research Square.

- Line 242 - Table 3 and Figure 2 - directionality needs to be made substantially clear in text, table and figures to indicate whether unregulated refers to up in RHD and down in ARF or vice-a-versa.

Response: The line "DEGs obtained in group X vs. group Y indicate genes with altered regulation in group X using group Y as a reference." has been added to the manuscript on pg 11. For example, RHD vs ARF indicates results obtained in RHD when compared to ARF. Thus, upregulated and downregulated in this context indicates genes upregulated and downregulated in RHD when compared to ARF i.e., considering ARF as the reference. The directionality has been made clearer in the text, tables, and figures as well.

- Line 245 - remove "In addition to the well-known ones" and describe genes without assumption about which are or are not well-known by readers

Response: We have removed this line in the revised manuscript.

- Line 264 - all network Figures must be provided in a high-resolution format that is easily readable

Response: We have provided high-resolution figures in the revised submission. The network figures for each comparison group have now been indicated separately, with the genes of interest (DEGs) labeled clearly for ease of visualization.

- Line 303 - further justify statement about similarities between conditions - e.g. chemokine signalling is mentioned in relation to RHD, ARF, and CHD

Response: We have added a note (pg 17), to describe similarities between conditions in the section - "Genome-wide perturbations present in RHD and ARF compared to Healthy controls". ARF and RHD are sequels to streptococcal infection and thus have an underlying inflammatory response that starts acutely in ARF and turns chronic in RHD. The inflammatory responses are regulated by chemokine, and cytokine signaling, such as increased production of which plays a role in CRP production, IL8, which is involved in chemotaxis, and TNFα which plays a role in increased production of pro-inflammatory cytokines, as well as chemotaxis in both the disease conditions. An increase in the production of these chemokines and cytokines eventually leads to valve damage. This condition also reflects in CHD. The current study's results also encapsulate these variations, indicating that the TopNets correctly capture the known immune responses in the disease conditions and their similarities.

- Line 321 - is the statement relating to the heatmap justified and are differences meaningful? although three RHD patients are neighbours the ARF patients appear scattered and the closest relationships appear to between different phenotypes. what parameters were used to draw this heatmap and how are they justified?

Response: The heat map was generated using the pheatmap software to hierarchically cluster the samples based on Z-scores scaled across the row (gene) for the 171 DEGs

identified as characteristic of RHD. Hierarchical clustering groups samples together iteratively without prior knowledge of the sample group (unsupervised). Samples with similar expression profiles and, in turn, similar phenotypes cluster together and are represented using a dendrogram, where the branches represent the distance or closeness between the samples. In this set as well, when annotated, the ARF and RHD samples fairly clustered well. Although a larger cohort of samples would provide a better understanding, there seems to be a reasonable level of similarity among the RHD samples represented in purple (3 out of the 5 samples clustered together) and ARF samples represented in green (4 out of the 5 samples clustered together) (pg 17-18).

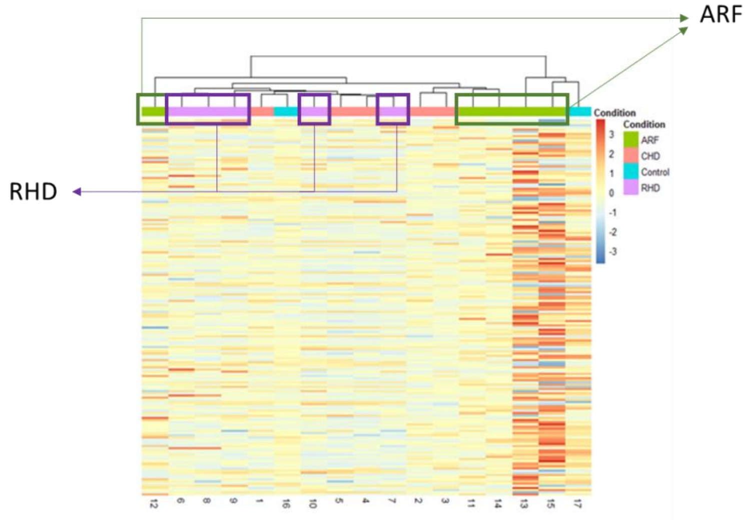

Discussion

Q- Line 357 - explain relationship between neutrophil-lymphocyte ratio to the wider transcriptome findings -- are there prior studies transcriptomic studies of the neutrophil-lymphocyte ratio in other traits that inform or contradict findings?

Response: As the neutrophil-lymphocyte ratio (NLR) is a simple and easily available clinical marker reflecting the general inflammatory index, it is prognostic in a variety of infections, cancers, and cardiovascular diseases. A high NLR was also identified in our study's ARF and RHD samples. The transcriptomics and network analyses carried out reflected the implication of this clinical finding where multiple proinflammatory genes, such as CXCL8 and CXCR6, were upregulated in RHD and ARF. Further pathway enrichment of the TopNets also captured Neutrophil extracellular trap formation as a significantly perturbed pathway (Supplementary file 2). A similar correlation between NLR and the transcriptomic profile of whole blood has been established in various other disease traits such as cancer, infectious diseases, and autoimmunity.

Please see the references below:

- **Papadopoulou G, Manoloudi E, Repousi N, Skoura L, Hurst T, Karamitros T. Molecular and Clinical Prognostic Biomarkers of COVID-19 Severity and Persistence. Pathogens. 2022 Mar 2;11(3):311. doi: 10.3390/pathogens11030311. PMID: 35335635; PMCID: PMC8948624.**
- **Maher MC, et al. Antibody therapy reverses biological signatures of COVID-19 progression. Cell Rep Med. 2022 Aug 16;3(8):100721. doi: 10.1016/j.xcrm.2022.100721. PMID: 35977462; PMCID: PMC9380250.**
- **Ming W, et al. Two Distinct Subtypes Revealed in Blood Transcriptome of Breast Cancer Patients With an Unsupervised Analysis. Front Oncol. 2019 Oct 1;9:985. doi: 10.3389/fonc.2019.00985. PMID: 31632916; PMCID: PMC6779774.**

- **Debien V, et al. Involvement of Neutrophils in Metastatic Evolution of Pancreatic Neuroendocrine Tumors. Cancers (Basel). 2021 Jun 2;13(11):2771. doi: 10.3390/cancers13112771. PMID: 34199561; PMCID: PMC8199674.**
- **Asano S, Chantler PD, Barr TL. Gene expression profiling in stroke: relevance of blood-brain interaction. CurrOpinPharmacol. 2016 Feb;26:80-6. doi: 10.1016/j.coph.2015.10.004. Epub 2015 Nov 9. PMID: 26562440; PMCID: PMC4716875.**
- **Lopez-Dominguez R, Toro-Dominguez D, Martorell-Marugan J, Garcia-Moreno A, Holland CH, Saez-Rodriguez J, Goldman D, Petri MA, Alarcon-Riquelme ME, Carmona-Saez P. Transcription Factor Activity Inference in Systemic Lupus Erythematosus. Life (Basel). 2021 Apr 1;11(4):299. doi: 10.3390/life11040299. PMID: 33915751; PMCID: PMC8065841.**

This information has been added on pg 19 of the manuscript.

Q- Line 366 - explain why up regulation of genes related to neutrophils is not a non-specific reflection of the acute state of patients enrolled in the study

Response: As mentioned above, considering that it was a small-scale study, we wish to reiterate that we selected the RHD cases stringently so as to avoid sampling patients with acute symptoms –as ascertained both clinically and using laboratory parameters (Pg 6, 7, 12). Hence, we think it is highly unlikely that the data presented in the manuscript is confounded by an acute state of the disease.

Q- Line 387 - relate text on TGF-beta to direction of up/down regulation in relation to RHD vs ARF - does this fit with the hypothesis?

Response: TGF-β signaling pathway was a major pathway perturbed in our analysis. 10 out of the 171 DEGs we found unique to RHD vs ARF are involved in TGF-β signaling, out of which ID3, RGMB, TNF, ACVR2B, SMAD7 were upregulated, and INHBA, BMPR1B, THBS1, BMP7, INHBE were downregulated. Although some of these are positive regulators, some negative regulators of TGF-β signaling seem to be upregulated as well, indicating some amount of downregulation of the pathway in RHD. This may indicate that the anti-inflammatory TGF-β signaling in the peripheral blood leukocytes that is generally upregulated in ARF when compared to healthy individuals may reduce upon progression to RHD. In essence, the reduced anti-inflammatory activity of the peripheral blood leukocytes observed in RHD in our study may contribute to the proinflammatory process in the damaged valves as evidenced by reduced TGF-β and increased TNF alpha signaling in this disease state. Increased expression of TGF-β and the downstream signaling pathway has been demonstrated in cardiac valves in RHD both in humans and experimental animals (Kim L et al., 2008; Zhao Z et al., 2020; Xian S et al., 2020). Thus, a crucial role of augmented secretion of TGF-β locally in the cardiac valves by the activated tissue macrophages steering upregulated TGF-β signaling in the valvular interstitial cells during ARF has been hypothesized to aid in the progression of the disease to RHD. Hence, in a nutshell, TGF-β signaling in the lesion is envisaged to initiate and sustain the fibroinflammatory process resulting in endothelial to mesenchymal transformation, thus promoting fibrosis and increased stiffness of the valves (Karthikeyan et al., 2020), and peripheral blood leukocytes may be aiding the proinflammatory process. Overall, our results point to a scenario of complex remodulation of the TGF-β signaling pathway occurring in the peripheral blood leukocytes, which may promote inflammation in the valves, most of the perturbations happening upstream of the pathway (Please see the KEGG image below with the DEGs marked in pink). Analysis in larger sample size would aid in clearly profiling the trend of expression of the pathway and its effect on ARF progression to RHD. This discussion has been added to the manuscript on pg 21-22.

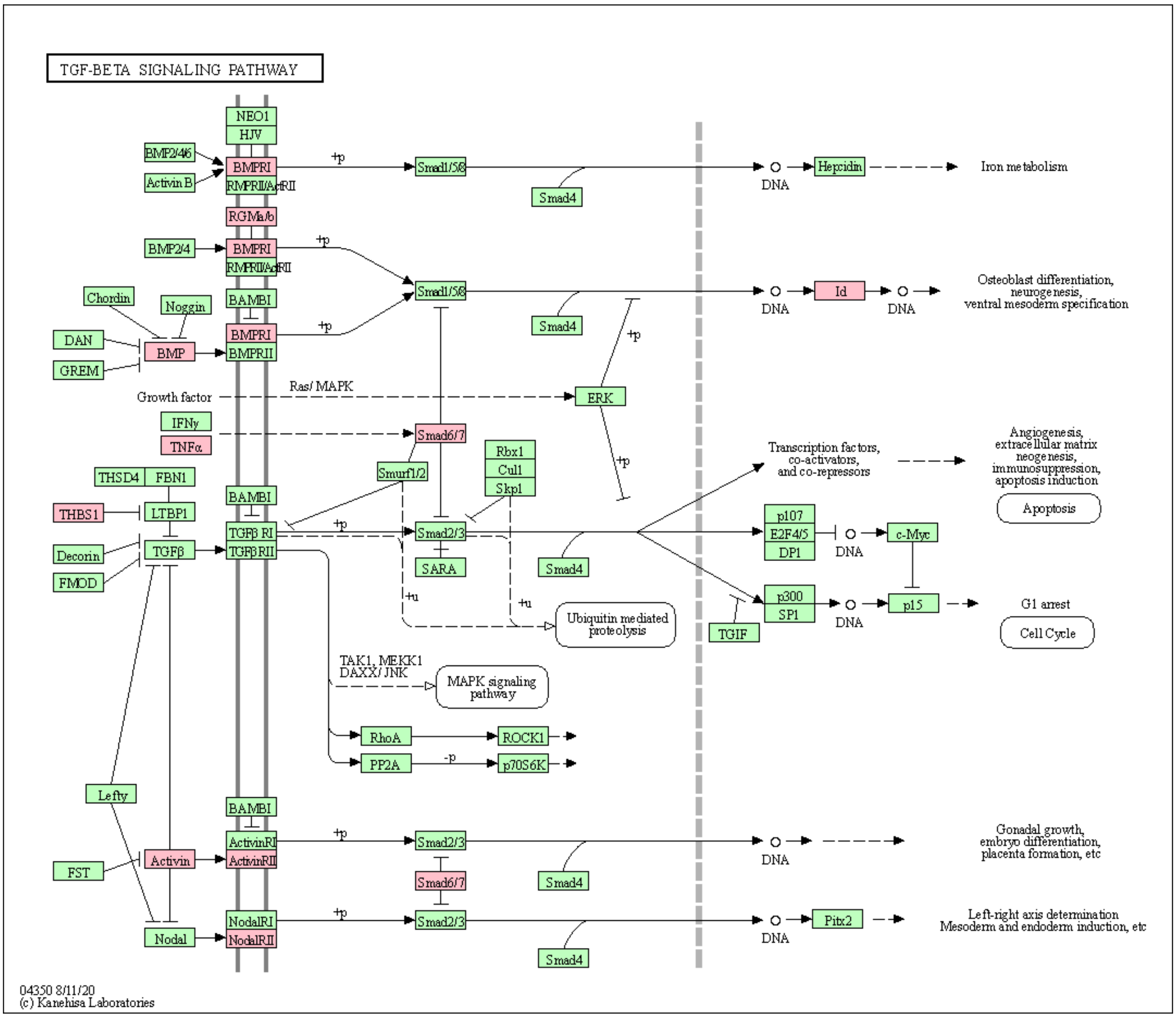

Kim L, Do KK, Woo IY, Dong HS, Ick MJ, Han KP, et al. Overexpression of Transforming Growth Factor-β1 in the Valvular Fibrosis of Chronic Rheumatic Heart Disease. J Korean Med Sci. 2008;23: 41. doi:10.3346/JKMS.2008.23.1.41

Zhao Z, He D, Ling F, Chu T, Huang D, Wu H, et al. CD4 + T cells and TGFβ1/MAPK signal pathway involved in the valvular hyperblastosis and fibrosis in patients with rheumatic heart disease. Exp Mol Pathol. 2020;114. doi:10.1016/J.YEXMP.2020.104402

XIAN S, CHEN A, WU X, LU C, WU Y, HUANG F, et al. Activation of activin/Smad2 and 3 signaling pathway and the potential involvement of endothelial-mesenchymal transition in the valvular damage due to rheumatic heart disease. Mol Med Rep. 2021;23. doi:10.3892/MMR.2020.11648

Karthikeyan, G., Fung, E. and Foo, R.S.Y., 2020. Alternative hypothesis to explain disease progression in rheumatic heart disease. Circulation, 142(22), pp.2091-2094.

Q- Line 396 - clarify reference 47

Response: Lower plasma levels of tryptophan and its catabolites have been reported in patients with ARF having recurrent streptococcal infections (Kurup and Kurup (2003). Low plasma concentrations of the amino acids could be interpreted to either be due to lower synthesis and/or higher catabolism. Considering that RHD is an extension of ARF, and that genes for tryptophan biosynthesis were downregulated in RHD compared to ARF in the present study, this reference was made.

Q- Line 407 - C4A is predominantly expressed in the liver -- please clarify the implications of this for the results

Response: Yes, we do agree that liver is the predominant source of component C4A of complement. However, peripheral blood leucocytes are known to be extrahepatic sites of synthesis of C4A (Ji E, et al., 2021; Melbourne JK, et al., 2018) The observation of increased expression of C4A in our study could be a reflection of the changes that occur systemically in this condition.

Ji E, et al., Peripheral complement is increased in schizophrenia and inversely related to cortical thickness. Brain Behav Immun. 2022 Mar;101:423-434. doi: 10.1016/j.bbi.2021.11.014. Epub 2021 Nov 19. PMID: 34808287

Melbourne JK, et al. C4A mRNA expression in PBMCs predicts the presence and severity of delusions in schizophrenia and bipolar disorder with psychosis. Schizophr Res. 2018 Jul;197:321-327. doi: 10.1016/j.schres.2018.01.018. Epub 2018 Feb 12. PMID: 29449061; PMCID: PMC6087677.

Summary and General Comments

Use this section to provide overall comments, discuss strengths/weaknesses of the study, novelty, significance, general execution and scholarship. You may also include additional comments for the author, including concerns about dual publication, research ethics, or publication ethics. If requesting major revision, please articulate the new experiments that are needed.

Reviewer #1: The present article is well written and interesting. The authors describe the identification of a sub network comprising the most significantly and differentially expressed genes and most perturbed pathways in RHD compared to ARF.
They evaluated the transcriptoma of 5 patients presenting ARF and 5 presenting RHD and identified a subnetwork comprising 53 significantly differentially expressed genes and most perturbed pathways in RHD compared 54 to ARF.
Figure 1, describe the overview of the article and is well presented.

Reviewer #2: This is a useful analysis in a neglected area but it should be presented as a pilot with very cautious results --- I would suggest the word "pilot" is added to the title. There needs to be much greater effort to identify key messages from the various comparisons made together are informative as well as indicate which of these findings are the most statistically robust given the multiple testing. It is vital that substantially greater clarity is added to the direction of effects and mandatory that the figures are presented in an updated high resolution readable format.

Response: The word “pilot” has been added to the title. The other concerns have also been addressed in the manuscript and high-resolution figures have been provided.
