# Supplementary material for: Profiling system-wide variations and similarities between Rheumatic Heart Disease and Acute Rheumatic Fever–A pilot analysis
Source: PLoS Negl Trop Dis. 2023 Apr 5;17(4):e0011263. doi: 10.1371/journal.pntd.0011263 (PMC10109489; doi:10.1371/journal.pntd.0011263)
Supplement: Attachment — Submitted filename: Manuscript PNTD 2022-01009.docx [file pntd.0011263.s003.docx]

**Manuscript PNTD-22-01009**

**Profiling system-wide variations and similarities between Rheumatic Heart Disease and Acute Rheumatic Fever**

The present article is well written and interesting. The authors describe the identification of a sub network comprising the most significantly and differentially expressed genes and most perturbed pathways in RHD compared to ARF.

They evaluated the transcriptoma of 5 patients presenting ARF and 5 presenting RHD and identified a subnetwork comprising 53 significantly differentially expressed genes and most perturbed pathways in RHD compared 54 to ARF.

**Figure 1**, describe the overview of the article and is well presented.

**Figure 2**, however is more difficult to follow as it show red nodes that represent genes in the top active 265 network, and also blue nodes that represent nodes of the top repressed network, and the purple nodes 266 indicate nodes common to both. Upper facing triangles represent genes that are upregulated, 267 and lower facing triangles indicate nodes that are downregulated. Functional enrichment of 268 some hub nodes such as GATA2, CCR3, SMAD7, and ALOX15 are also shown.

As a reviewer, I suggest the authors to amplify this Figure, that will facilitate the reader and to add value to the article.
